# Supplementary material for: Dynamic Surface Topography for Thoracic and Lumbar Pain Patients—Applicability and First Results
Source: Bioengineering (Basel). 2025 Mar 13;12(3):289. doi: 10.3390/bioengineering12030289 (PMC11939185; doi:10.3390/bioengineering12030289)
Supplement: Supplementary file 1 [file bioengineering-12-00289-s001.zip › bioengineering-3481105-supplementary.pdf]

Supplementary Table S1: Effect sizes of the sum of motion per gait cycle (SoMpGC)

speed	VP/ T1	T1/ T2	T2/ T3	T3/ T4	T4/ T5	T5 /T6	T6/ T7	T7/ T8	T8/ T9	T9/ T10	T10/ T11	T11/ T12	T12/ L1	L1/ L2	L2/ L3	L3/ L4	L4/ Pelvis
2km/h	0.7	0.7	0.8	0.9	0.9	0.6	0.2	0.1	0.3	0.5	0.7	0.8	0.4	0.4	0.5	0.5	0.3
3km/h	0.8	0.8	0.7	0.7	0.7	0.4	-0.3	0.4	0.5	0.1	0.1	0.2	0.1	0.3	0.4	0.3	0.2
4km/h	0.7	0.6	0.6	0.6	0.5	0.3	-0.3	0.4	0.6	0.4	0.2	0.3	0.0	0.1	0.2	0.2	0.1
5km/h	0.4	0.4	0.1	0.0	0.0	-0.1	-0.1	0.3	0.2	-0.2	-0.3	-0.2	-0.2	-0.1	0.0	-0.1	-0.1

Caption: displayed are the effect sizes for all segments of the sum of motion per gait cycle (SoMpGC) for all speeds. Positive values points towards the painful area. negative values points towards the healthy reference groups
